# The Added Value of Bronchoalveolar Lavage for Pulmonary Tuberculosis Diagnosis in High-Risk Hospitalized Patients with Negative Sputum Samples

**DOI:** 10.3390/arm92010003

**Published:** 2023-12-21

**Authors:** Ophir Freund, Yitzhac Hadad, Tomer Lagziel, Inbal Friedman Regev, Eyal Kleinhendler, Avraham Unterman, Amir Bar-Shai, Tal Moshe Perluk

**Affiliations:** 1 Institute of Pulmonary Medicine, Sourasky Medical Center, Faculty of Medicine, Tel-Aviv University, Tel-Aviv 6423906, Israel; inbal08@gmail.com (I.F.R.); amirbs@tlvmc.gov.il (A.B.-S.);; 2 Radiology Department, Sourasky Medical Center, Faculty of Medicine, Tel-Aviv University, Tel-Aviv 6423906, Israel; 3 Department of Surgery, Mount Sinai Hospital, Icahn School of Medicine at Mount Sinai, New York, NY 10029, USA

**Keywords:** tuberculosis, diagnosis, bronchoscopy, hospital, sputum, bronchoalveolar lavage

## Abstract

**Highlights:**

**What are the main findings?**

- BAL was a diagnostic for pulmonary TB in 7% of high-risk isolated inpatients with negative sputum samples.
- Upper-lobe consolidation in chest X-rays, ≥2 sub-acute symptoms, and centrilobular nodules in chest CT scans were among the independent predictors for pulmonary TB.

**What is the implication of the main finding?**

- Negative sputum samples had relatively good predictive ability in patients hospitalized with suspicion of pulmonary TB.
- Assessing sociodemographic, clinical, and radiological attributes could improve patient selection and reduce unnecessary tests and costs.

**Abstract:**

Hospitalized patients with a high suspicion of pulmonary tuberculosis (HS-PTB) are isolated until a definite diagnosis can be determined. If doubt remains after negative sputum samples, bronchoscopy with bronchoalveolar lavage (BAL) is often sought. Still, evidence of the added value of BAL in this patient population is scarce. To address this issue, we included consecutive HS-PTB patients with negative sputum samples who underwent BAL between 2017 and 2018. Chest X-rays (CXR) and CT scans were evaluated by a chest radiologist blind to the final diagnosis. Independent predictors for PTB were assessed by multivariate regression, using all positive PTB patients between 2017 and 2019 (by sputum or BAL) as a control group (*n* = 41). Overall, 42 HS-PTB patients were included (mean age 51 ± 9, 36% female). BAL was a viable diagnostic for PTB in three (7%) cases and for other clinically relevant pathogens in six (14%). Independent predictors for PTB were ≥2 sub-acute symptoms (adjusted OR 3.18, 95% CI 1.04–9.8), CXR upper-lobe consolidation (AOR 8.70, 95% CI 2.5–29), and centrilobular nodules in chest CT (AOR 3.96, 95% CI 1.20–13.0, *p* = 0.02). In conclusion, bronchoscopy with BAL in hospitalized patients with HS-PTB had a 7% added diagnostic value after negative sputum samples. Our findings highlight specific predictors for PTB diagnosis that could be used in future controlled studies to personalize the diagnostic evaluation.

## 1. Introduction

Tuberculosis (TB) is a highly prevalent infectious disease, with *Mycobacterium tuberculosis* (mTB) as the causative pathogen that has infected one-third of the world’s population [1]. In general, infection with TB divides into latent and active forms. Latent TB is the presence of infection without clinical symptoms, radiological abnormality, or microbiological evidence [1]. Pulmonary tuberculosis (PTB) is caused when TB infects the lungs and results in active disease. It is the leading cause of death worldwide from a single infectious agent [2]. PTB is suspected based on a combination of clinical, radiological, and epidemiological features. Clinically, PTB can manifest with prolonged coughing, hemoptysis, lymphadenopathy, night sweats, and periodic fevers [3]. Epidemiologically, among the high-risk factors are prior exposure to TB, history of prior TB, and residing in/travelling to endemic areas [4]. Those who meet the clinical and epidemiological criteria undergo chest imaging as an initial work-up, mainly with chest CT scans [5]. Radiological features of PTB change with disease evolvement. Primary PTB is characterized by hilar and mediastinal nodes with central hypo-dense areas on enhanced CT. Post-primary disease, which is the usual presentation in adults, is mainly characterized by cavitation or poorly defined consolidation in the apical and posterior segments of the upper lung lobes [6]. Patients presenting to the hospital with high suspicion for PTB (HS-PTB) based on the above factors and requiring hospitalization are isolated in negative-pressure rooms until the diagnosis is confirmed or treatment commences.

Making a correct and fast diagnosis of PTB has major implications for both the patient and public health in general. This is also highlighted by the “End TB” strategy led by the WHO organization [7]. As the diagnostic evaluation could take time and resources, it is vital to correctly identify patients at risk of PTB. The main clinical practice guidelines regarding the diagnosis of TB from 2017 do not present specific criteria for determining who is at high risk of PTB [5]. Rather, they recommend weighing the level of exposure to TB (as an indicator of risk of infection) with clinical data such as comorbidities and chest X-rays (as indicators for risk of developing active disease). More specific tools were developed to identify high-risk people for PTB. For example, the four symptoms screen (current cough, fever, weight loss, or night sweats) was found to have high sensitivity to identify at-risk people among those living with human immunodeficiency virus (HIV) [8]. Another study by Li et al. included 14,044 household contacts of adults with PTB to derivate and validate two clinical prediction tools for high-risk people [9]. In both tools, a cough of over 1–2 weeks was the single strongest factor for prediction. These studies highlight the difficulty in diagnosing PTB in patients with mild or no symptoms. While there is a growing amount of research in this field, there are still no widely accepted criteria to determine people at high risk of PTB.

For the diagnosis of PTB, sputum smears for acid-fast bacillus (AFB) are often considered the first test of choice [5,10]. However, sputum microscopy has only approximately 70% sensitivity for diagnosis. Adding cultures and nucleic acid amplification tests to sputum specimens leads to a significantly higher diagnostic yield, but patients are still being diagnosed with PTB after having negative sputum samples [10,11]. In addition, many patients are unable to expectorate sputum. In these cases, several procedures were shown to be effective, such as induced sputum or supervised sputum production, especially among patients with limited disease or minimal symptoms [12,13]. Still, patients unable to expectorate sputum present a major barrier to PTB diagnosis. When non-invasive sputum production fails or remains negative for TB, flexible bronchoscopy with bronchoalveolar lavage (BAL) is the next diagnostic test, as recommended by the main guidelines [5,14]. Bronchoscopy has been shown to have a high accuracy in ruling out active PTB [15]. However, the added diagnostic value of BAL in HS-PTB cases remains unknown. A limited number of studies reported the yield of BAL in this patient population, and their results showed high variability of diagnostic performances [16,17,18,19]. Among these studies, only a few included consecutive patients, were conducted in the in-patient setting, or used cohorts of patients with other final diagnoses to understand its added diagnostic value. Not surprisingly, the recommendation by the American Thoracic Society (ATS) guidelines for the use of BAL is only supported by a “very low-quality of evidence” [5].

Considering the above, our aim was to demonstrate the added diagnostic value of flexible bronchoscopy with BAL among consecutive HS-PTB hospitalized patients with negative sputum samples in a real-life setting. We also aimed to understand the predictors for a final diagnosis of PTB in this patient population to enable better patient selection for this procedure in the future.

## 2. Materials and Methods

### 2.1. Study Design and Population

This retrospective observational study included all consecutive hospitalized adult (age > 18) patients who underwent bronchoscopy with BAL in a large tertiary medical center between 1 January 2017 and 1 December 2018. In general, as part of the common practice in our medical center, patients who are not able to expectorate sputum undergo sputum induction at least once before making the decision to perform a bronchoscopy. In cases of negative or unavailable sputum samples, the decision to perform bronchoscopy with BAL for additional testing is made by a consultation of the treating physicians, a senior infectious disease specialist, and a senior pulmonologist. This decision is made only for patients considered to have HS-PTB by the mentioned personnel based on known risk factors and a negative investigation of other infectious and non-infectious etiologies. Patients with a relatively low suspicion for PTB are able to stop isolation and continue with other non-PTB-related evaluations. Patients with high suspicion remain in isolation and proceed to bronchoscopy, usually as a final exclusion test.

Subjects included in our final cohort had to meet the following criteria: (1) hospitalization for active symptomatic pulmonary disease suspected to be PTB, (2) validation by the research team of HS-PTB (defined below), (3) undergoing bronchoscopy with BAL for the diagnosis of PTB during admission, and (4) having at least one negative sputum sample before BAL. Patients with unavailable access to their medical records from hospitalization (for example, due to confidential data) were excluded.

HS-PTB patients were defined as having all of the following: (1) high clinical suspicion by the treating physician, reflected by patient isolation during admission in a negative pressure room, (2) one or more PTB-related sub-acute symptom or imaging finding (consolidation or cavitation), and (3) one or more risk factor for PTB (such as household contact, immigrants from high-burden countries, intravenous drug users, and immunosuppression) [5,20]. Sub-acute symptoms related to TB included hemoptysis, night sweats (more than 2 episodes of heavy sweating during sleep in the month prior to hospitalization), a cough for at least two weeks, unintentional weight loss (>5 kg in the past 6 months), pleuritic chest pain, or recurrent fevers (>38 °C, at least once a week for more than 2 weeks).

### 2.2. Ethical Statement

The study was approved by the Tel-Aviv Medical Center institutional review board (IRB number 0008-19-TLV, approval date 8 January 2019) and conducted in accordance with the declaration of Helsinki. Informed consent was waived because of the retrospective design of the study. The study was performed according to STROBE guidelines.

### 2.3. Predictors for Pulmonary TB Diagnosis

To evaluate predictors for a final diagnosis of PTB, we analyzed all patients who were diagnosed with PTB during hospitalization based on positive culture or polymerase chain reaction from sputum samples or BAL between 1 January 2017 and 1 December 2018. This group of patients is defined as the control group and was compared with patients having negative BAL from our cohort to assess the predictors for PTB diagnosis. The final diagnosis of each patient in our cohort and the control group was determined by the treating physicians with consultations from senior infectious diseases specialists and pulmonologists during admission and was validated by a senior pulmonologist for the purpose of this study (TMP).

### 2.4. Data Collection

Baseline variables (age, sex, comorbidities, demographics, and risk factors for PTB) and clinical variables (symptoms, prior and current cultures, sputum samples, imaging, and BAL results) were extracted from electronic hospital records. For this purpose, we reviewed all admission and discharge letters, follow-up notes during admission, and any visits to the respiratory or infectious diseases clinics. All included patients in our cohort had available chest CT scans that were performed during their admission (in the control group, 6 patients did not perform a chest CT scan). CT images were acquired using a 128 × 2 × 0.625 mm detector rows scanner (ICT 256; Philips Healthcare, Cleveland, OH, USA). Additional acquisition parameters were tube voltage of 120 kV, pitch of 1.2, matrix = 512 × 512, rotation time ranging 300 ms, and slice thickness = 1.0–1.5 mm. Images were reconstructed with a slice of 1.0–2.0 mm with the same increment. A radiologist specializing in chest imaging (YH) reviewed all chest X-rays (CXR) and CT scans and evaluated the presence of consolidations and cavitation. Centrilobular nodules and lymphadenopathy were also assessed in the CT scans. The radiologist was blind to patients’ medical data, including the final diagnosis. For analysis, immunosuppression was defined as active hematologic malignancy, active HIV, or treatment with immunosuppressive drugs (steroids were considered immunosuppression if administered for more than one month in an equivalent dose of 10 mg prednisone).

### 2.5. Procedures and Laboratory Analysis

Bronchoscopy was performed using standard methods and guidelines [14]. BAL was performed after the patient had fasted overnight; local anesthesia and sedation were administered, and the procedure involved inspecting the bronchial tree, instilling 100–300 mL of normal saline, followed by aspiration of BAL fluid from affected lung segments indicated by prior chest imaging. Samples were processed with at least 10 mL of BAL fluid.

Sputum and induced sputum samples were collected according to accepted guidelines [21]. In general, they were the first morning samples, with at least two samples sent for AFB smear and MTB culture. Patients were followed by a nurse and asked to cough several times after rinsing their mouths with water and expectorate into a sterile container. Induction was performed using inhalation of 3% hypertonic saline. Our institution laboratory performed Ziehl-Neelsen staining and MTB culture (both solid and liquid media) tests on a routine basis for any suspected TB case. The polymerase chain reaction (PCR) using the GeneXpert MTB/RIF assay was performed on a case-by-case basis according to infectious diseases consultation.

### 2.6. Statistical Analysis

Categorical variables were presented as totals (percentages) and compared with Chi-square tests. Normally distributed continuous variables were presented as mean ± standard deviation (SD) and compared with unpaired *t*-tests. Non-normally distributed continuous variables were presented as a median (interquartile range, IQR) and compared with Mann–Whitney U tests. Normal distribution was assessed using the Kolmogorov–Smirnov test. Independent predictors for PTB diagnosis were analyzed with multivariate logistic regression models using backward conditional elimination and including the significant variables from univariate analyses and clinically relevant variables. We used two models, one for all patients and another that included CT-related variables that were missing for 6 patients. Data were analyzed using IBM SPSS Statistics for Windows, Version 28.0, and *p* < 0.05 was determined for significance.

## 3. Results

**Figure 1:**
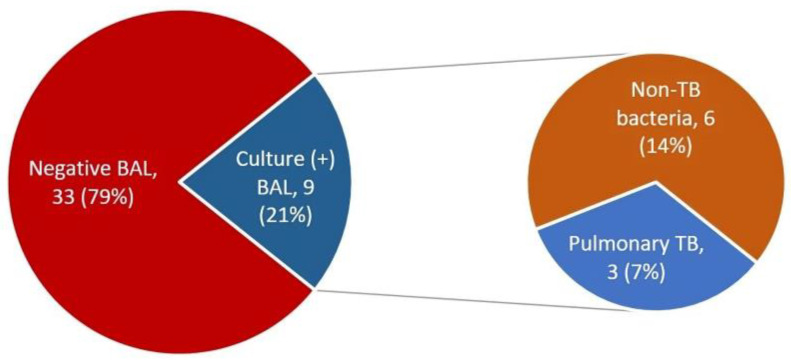
Results of bronchoalveolar lavage (BAL) among the study cohort.

During the study period, 45 patients with HS-PTB underwent bronchoscopy with BAL; of them, three were not able to expectorate sputum before the exam (including after sputum induction) and were excluded from our cohort. All patients had available medical records. Of the 42 included patients, 3 (7%) had positive BAL for TB, and 6 (14%) had positive BAL cultures for other pathogens that were considered to be clinically relevant and received treatment (Figure 1).

### 3.1. Study Cohort

The cohort’s mean age was 51 ± 9; 36% were females, 57% had immunosuppression, and 67% were immigrants (Table 1). Immunosuppression was caused by infection with HIV in 14 cases and immunosuppressive treatment in 10 cases (7 with hematological malignancy and 3 with inflammatory bowel disease). Of note, all hematological malignancies were already diagnosed and undergoing treatment initiation at the time of admission. The origins of immigrants included Africa (13 subjects), eastern Europe (12 subjects), and South America (3 subjects). None of the patients had prior TB or received prior treatments for TB.

Overall, 62% had at least one TB-related sub-acute symptom, and 33% had more than two. The most common symptom was recurrent fevers in 15 cases (36%). The median (IQR) time from symptoms to hospitalization was 3.5 weeks (2.5–7.5). Among laboratory results (first samples from hospital arrival) were a median hemoglobin of 12 g/dL (normal range 11.7–15.5), leukocytes of 10.1 K/uL (normal range 4–11), c-reactive protein of 55 mg/L (normal range 0–5), and albumin of 34 g/L (normal range 35–50).

Findings in CXR included consolidations (45%) and cavities (7%). The main chest CT features included consolidations (55%), a higher rate of cavities (24%), and centrilobular nodules (33%). Most consolidations in CXR (52%) and CT (69%) were in the upper lobes.

### 3.2. Predictors for Pulmonary TB

The control group (*n* = 41) was comprised of hospitalized patients diagnosed with pulmonary TB by sputum (*n* = 38) or by BAL (from our initial cohort, *n* = 3). Therefore, for comparison, our cohort included 39 patients with negative BAL results. Of note, two patients with positive sputum for TB had unavailable medical records and were therefore excluded. Patients in the control group were younger (mean age 39 vs. 52, *p* < 0.01) and had a lower rate of immunosuppression (17% vs. 59%, *p* < 0.01). They had higher rates of people with any TB-related symptoms (88% vs. 62%, *p* < 0.01) and with >2 related symptoms (66% vs. 36%, *p* < 0.01). The control group also had a greater total amount of PTB-related sub-acute symptoms (median 2, IQR 1–3, vs. median 1, IQR 0–2, *p* < 0.01).

Findings in CXR were more prevalent among the control group, including cavitation (32% vs. 8%, *p* < 0.01) and upper lobe consolidation (71% vs. 18%, *p* < 0.01). Similarly, the control group (*n* = 35, 6 missing a CT scan) had higher rates of CT findings, including upper lobe consolidation (71% vs. 36%, *p* < 0.01) and centrilobular nodules (60% vs. 33%, *p* = 0.02).

Table 2 presents the two multivariate models, first for the entire cohort (general model) and second for patients with available chest CT scans from their hospitalization (radiological model). In the general model, independent predictors for PTB diagnosis were two or more sub-acute symptoms (adjusted OR 3.18, 95% CI 1.04–9.8, *p* = 0.04) and upper lobe consolidation in CXR (AOR 8.70, 95% CI 2.5–29, *p* < 0.01). In contrast, independent predictors for negative PTB diagnosis were age above 50 (AOR 0.11, 95% CI 0.03–0.42, *p* < 0.01) and female sex (AOR 0.25, 95% CI 0.06–0.94, *p* = 0.04). In the radiological model, the presence of upper lobe consolidation in chest CT scans (AOR 7.37, 95% CI 2.06–26.5, *p* < 0.01) and centrilobular nodules (AOR 3.96, 95% CI 1.20–13.0, *p* = 0.02) were also independent predictors for PTB.

### 3.3. Additional Diagnoses by BAL

Other than the three positive BAL samples for TB, six additional tests were positive for other bacteria deemed clinically relevant: *Pneumocystis jirovecii* (*n* = 1), *Staphylococcus aureus* (*n* = 2), *Pseudomonas aeruginosa* (*n* = 2), and *Serratia marcescens* (*n* = 1). One patient was treated as having PTB based on high suspicion, which remained after the negative bronchoscopy. All other patients (*n* = 32, 76%) were treated with empirical antibiotics and did not have a diagnosis of TB in their follow-up after discharge.

## 4. Discussion

In this study, we evaluated the added diagnostic value of bronchoscopy with BAL among hospitalized patients with negative sputum samples. As a routine practice in our medical center, only patients with HS-PTB undergo BAL in cases of negative sputum samples, which was also validated for this study by the research team. We found that using BAL led to a diagnosis of PTB in 7% of the cohort, with an overall added diagnostic value of 21% (identified clinically relevant pathogen). The number of TB-related symptoms was significantly higher among patients with positive PTB. Among patients with HS-PTB, younger age, more than two PTB-related symptoms, upper lobe consolidation in CXR, and centrilobular nodules on chest CT were independent predictors for PTB.

Deciding which patients with HS-PTB and negative sputum samples should undergo bronchoscopy is of high importance. Bronchoscopy with BAL, although generally safe, still holds a rate of approximately 15% complications, which are mainly self-limiting hypoxemia [22]. In addition, bronchoscopy has high costs without proven greater effectiveness [23,24] and is often not available in countries with the highest prevalence of PTB [25]. Our aim was to assess the value of BAL in a real-world scenario. The few studies addressing this issue mainly include patients with a known final diagnosis of PTB and look at the diagnostic yield of bronchoscopy among them [18,26,27]. This method of selection can induce selection bias, underlined by the studies’ high diagnostic yield of bronchoscopy (57–83%), and does not reflect real-life decision-making for patients hospitalized with HS-PTB. In addition, the added value of BAL, specifically in hospitalized patients with HS-PTB, is unknown and was the main aim of our study.

We found an added value of 7% for PTB diagnosis in our cohort. Iyer et al. examined the role of BAL in HS-PTB patients and found a similar diagnostic yield of 10% [19]. In contrast, Ahmad et al. found 32% added diagnoses of PTB in a similar cohort of patients [16]. Both of the above studies and others have included non-hospitalized patients with positive screening tests, which could increase the pre-test probability of PTB compared with our cohort [28]. To improve the general diagnostic yield, post-bronchoscopy sputum specimens should be collected as they were shown to result in over 4% additional diagnoses and are recommended by the ATS guidelines [5,29]. Other innovative approaches that show promise in different pulmonary diseases, such as nanoparticle analysis from sputum or whole genome sequencing [30,31], could provide better alternatives for diagnosis in the future.

Our results regarding the added diagnostic yield of BAL could be explained in two ways or be the result of the combination of the two. First, it might be that patient selection for BAL was substandard in our cohort, leading to a lower diagnostic yield. The multidisciplinary shared decision to pursue BAL and the high rates of TB-related risk factors are against this hypothesis. On the other hand, higher rates of CT findings compared with CXR in our cohort could imply milder or smaller radiologic features than the control group, hence sub-optimal patient selection for BAL. Regardless, we hope to improve future patient selection with our analysis of predictors for PTB. The second explanation for the diagnostic yield could be a high negative predictive value of sputum samples. Prior studies found a similar diagnostic yield between sputum induction and BAL, highlighting the value of high-quality sputum samples in terms of final PTB diagnosis [32,33].

Predictors for PTB in our cohort should be separated from those in the general population, as included subjects had high clinical suspicion and active symptoms. For example, we found a large difference in age between the groups, with significantly younger patients among those diagnosed with PTB. Although ages between 55–64 are known to be a risk factor in the general population, and even among immigrants [34,35], younger patients presenting to the hospital with relevant risk factors, symptoms, and imaging findings are less likely to have other etiologies such as non-resolving pneumonia or abscesses, hence having a higher likelihood of PTB. Similarly, patients with immunosuppression are obviously at higher risk for PTB in the general population, while high-risk hospitalized patients are more likely to have other atypical infections than immunocompetent patients with similar high-risk clinical findings [22]. This could explain the significant differences in rates of immunosuppression between HS-PTB patients with negative BAL and those with a diagnosis of PTB. Finally, it seems that TB-related sub-acute symptoms and imaging findings should have a greater impact on the decision to pursue PTB diagnosis by BAL. Upper zone lung abnormalities and centrilobular nodules were previously shown to correlate with positive sputum, enforcing our results [16,36].

Our study has limitations. Subjects were included from a single tertiary center, and both generalizability and referral bias could not be excluded. Although we included consecutive patients, patients were referred to bronchoscopy at the discretion of their treating physician, infectious disease specialists and pulmonologists, and a selection bias is possible. Whether sputum was obtained by induction or not was not available for some of the patients and, therefore, was beyond the scope of our study. All sputum and BAL samples were sent for Ziehl-Neelsen stain and mTB cultures, while PCR was available for only some of the patients. However, none of the patients had a change in the diagnosis of PTB according to any follow-up visit or hospitalization in the following year.

## 5. Conclusions

Bronchoscopy with BAL in hospitalized patients with HS-PTB had an added diagnostic value of 7% of PTB and 14% of other clinically meaningful pathogens. Whether the diagnostic yield was affected by a high negative predictive value of sputum samples or sub-optimal patient selection is uncertain. We believe that BAL still has an important place in the diagnostic evaluation of PTB for a selected patient population, although future prospective controlled studies are required to make specific recommendations.

## Figures and Tables

**Table 1:** Study cohort characteristics.

Variable	Study Cohort, *n* = 42 (%)
Age	51 ± 9
Female sex	15 (36)
Immunocompromised	24 (57)
Known exposure	10 (24)
Immigrants	28 (67)
Symptoms	
Chronic cough	14 (33)
Haemoptysis	5 (12)
Recurrent fevers	15 (36)
Night sweats	7 (17)
Weight loss	9 (21)
Pleuritic chest pain	2 (5)
TB-related subacute symptoms	26 (62)
≥2 symptoms	14 (33)
Laboratory variables ^a^	
Hemoglobin (g/dL)	12 (10.7–13)
Leukocytes (K/uL)	10.1 (6.4–14.3)
Neutrophils (K/uL)	6.45 (2.9–10.1)
Sodium (mmol/L)	137 (133–139)
C-reactive protein (mg/L)	55 (24–109)
Albumin (g/L)	34 (32–38)
Imaging	
CXR—consolidation	19 (45)
CXR—upper lobe consolidation	10 (24)
CXR—cavitation	3 (7)
CT—consolidation	23 (55)
CT—upper lobe consolidation	16 (39)
CT—cavitation	10 (24)
CT—centrilobular nodules	14 (33)
CT—lung lymphadenopathy	17 (43)

CXR, chest X-ray. ^a^ First results from hospital arrival, data missing for 3 patients.

**Table 2:** Univariate and multivariate analysis for predictors of TB diagnosis.

Variable	Univariate		Multivariate	
	OR (95% CI)	*p*	AOR (95% CI)	*p*
A. General Model				
Age ≥ 50 years	0.22 (0.07–0.61)	<0.01	0.11 (0.03–0.42)	<0.01
Female sex	0.37 (0.13–0.17)	0.05	0.25 (0.06–0.94)	0.04
≥2 sub-acute symptoms	3.44 (1.37–8.63)	<0.01	3.18 (1.04–9.81)	0.04
CXR upper lobe consolidation	11.1 (3.83–27.9)	<0.01	8.70 (2.51–29.3)	<0.01
B. Radiological Model (N = 74) ^a^				
Age ≥ 50 years	0.26 (0.09–0.74)	0.01	0.12 (0.03–0.47)	<0.01
Female sex	0.45 (0.16–1.18)	0.13	0.30 (0.08–1.21)	0.09
≥2 sub-acute symptoms	2.68 (1.05–6.86)	0.04	2.15 (0.86–6.96)	0.104
CT upper lobe consolidation	4.46 (1.67–11.9)	<0.01	7.37 (2.06–26.5)	<0.01
Centrilobular nodules in CT	3.00 (1.16–7.75)	<0.01	3.96 (1.20–13.0)	0.02

^a^ Including patients that had a chest CT scan during admission (6 missing from the control group).

## Data Availability

All data generated or analyzed during this study are included in this article. Due to ethical and privacy concerns, the primary dataset cannot be made openly available. Requests for the dataset supporting our results can be made via helsinki@tlvmc.gov.il and will be granted by the first author after approval.
